# MOF-derived Co^2+^-doped TiO_2_ nanoparticles as photoanodes for dye-sensitized solar cells

**DOI:** 10.1038/s41598-021-95844-4

**Published:** 2021-08-11

**Authors:** R. Krishnapriya, C. Nizamudeen, B. Saini, M. Sayem Mozumder, Rakesh K. Sharma, A.-H. I. Mourad

**Affiliations:** 1 Mechanical Engineering Department, College of Engineering, United Arab Emirate University, Al Ain 15551, UAE; 2 Department of Chemistry, Indian Institute of Technology Jodhpur, Jodhpur, 342037 Rajasthan India; 3 Department of Chemical and Petroleum Engineering, College of Engineering, United Arab Emirate University, Al Ain, 15551 UAE; 4 National Water and Energy Centre, United Arab Emirate University, Al Ain, 15551 UAE; 5 On Leave From Mechanical Design Department, Faculty of Engineering, Helwan University, Cairo, Egypt

**Keywords:** Solar cells, Nanoparticles

## Abstract

Facile synthesis and application of nano-sized semiconductor metal oxides for optoelectronic devices have always affected fabrication challenges since it involves multi-step synthesis processes. In this regard, semiconductor oxides derived directly from metal–organic frameworks (MOFs) routes have gained a great deal of scientific interest owing to their high specific surface area, regular and tunable pore structures. Exploring the application potential of these MOF-derived semiconductor oxides systems for clean energy conversion and storage devices is currently a hot topic of research. In this study, titanium-based MIL-125(Ti) MOFs were used as a precursor to synthesize cobalt-doped TiO_2_-based dye-sensitized solar cells (DSSCs) for the first time. The thermal decomposition of the MOF precursor under an air atmosphere at 400 °C resulted in mesoporous anatase-type TiO_2_ nanoparticles (NPs) of uniform morphology, large surface area with narrow pore distribution. The Co^2+^ doping in TiO_2_ leads to enhanced light absorption in the visible region. When used as photoanode in DSSCs, a good power conversion efficiency (PCE) of 6.86% with good photocurrent density (*Jsc*) of 13.96 mA cm^−2^ was obtained with the lowest recombination resistance and the longest electron lifetime, which is better than the performance of the pristine TiO_2_-based photoanode.

## Introduction

The depletion of natural resources and environmental pollution have intensified climate change and outpaced nature's sustainability. Consequently, the advancement of sustainable clean energy technologies that are highly efficient and cost-effective is inevitable and getting innovative scientific attention^1–4^. Alternative energy technologies such as biomass, wind, wave, geothermal, and solar energies are highly desirable to compensate for the current energy demand scenario. Amidst the above-mentioned renewable energy sources, sunlight is clean and accessible at free cost. Solar cells are ingenious devices that can effectively harness and convert sunlight directly to useful electrical energy through the photoelectric effect^5–7^. Accordingly, solar photovoltaic (PV) technology appears very viable solution. Compared to other PV technologies, dye-sensitized solar cells (DSSCs) are prospective candidates owing to their simple solution-processable synthesis and cost-effective fabrication procedures^8,9^.

A DSSC device mainly consists of a photoanode with a wide bandgap semiconductor (TiO_2_, ZnO, SnO_2_, etc.) layer, a photosensitizer (dye), a redox electrolyte-based on I^−^/I_3_^−^ and a Platinum-based counter electrode (CE)^10–12^. Photoanodes are a crucial part of DSSC, and their functions include providing a high surface area to adsorb adequate dye molecules for effective light-harvesting, facilitating a suitable path for electron transport, and providing macropores for effective electrolyte diffusion. Thus, materials that possess intriguing features such as high surface area and tunable porosity, and other surface properties are prerequisites^13–18^. To improve the light harvesting efficiency (LHE) of photoanode, strategies like the incorporation of plasmonic nanoparticles of Ag and Au as well as the use of graphene and graphdiyne materials were reported recently^19–22^. The cell parameters, precisely, the open-circuit photovoltage (*V*_*OC*_), short-circuit current density (*J*_*SC*_), fill factor (FF), and the power conversion efficiency (PCE/*η*), are considerably affected by the microstructures of the photoanode materials^23,24^. Commonly used photoanode of DSSCS is TiO_2_ owing to its relatively high PCE, high surface area, non-toxicity, and low cost. However, random electron transport in this material results in the recombination of electron–hole pairs leading to poor device performance.

Moreover, the large bandgap of TiO_2_ (3.3 eV) often poses the proper excitation and injection of photoelectrons, leading to inept electron transportation and electrical conductivity. Bandgap engineering via doping with metals can moderate or reduce the optical band gap by introducing many intermediate energy levels in TiO_2_ and can cause feasible electron transitions with minimum excitation energy^25–27^. Such a doped semiconductor oxide layer demonstrated less recombination rate for the photogenerated electron and hole pairs with the advantage of many trapped states. This doping methodology is highly promising for semiconductor-based optoelectronic devices^28–30^. Among various metals reported for doping, transition metal (TM) doped TiO_2_ NCs have been ascertained as appropriate candidates for broad photoresponse in the visible light region and hence demonstrated as effective for solar energy conversion^31–33^.

Additionally, extensive research has been devoted to improve the photoanode properties of DSSCs, in which the introduction of porous photoanodes made of various template-assisted synthesis was successfully employed^34–38^. However, the broad applicability of these photoanodes were limited due to high fabrication cost and unfavorable possibilities for large scale production. Recently, mesoporous TiO_2_ materials derived from metal–organic frameworks (MOFs) are getting much attention owing to their advantages, like high porosities and extraordinary variability in structural engineering^39–41^. MOFs are a special class of porous materials formed of metal ions and organic linkers^42,43^. It can be applied as efficient templates to fabricate hierarchically porous metal oxide nanostructures for DSSC electrode application. These MOF-derived metal oxides often benefit from precise morphologies, huge porosities, easy methods to dope heteroatoms, and metal nanoparticles. They can exhibit significant electrochemical properties, which are intrinsically inherited from the precursor MOF used^39,44^. However, the application of MOF-derived TiO_2_ is not well explored for photovoltaic applications.

ZIF-8 is the first MOF that was applied for DSSC applications, where its thin layer was coated on the conventional TiO_2_ photoanode^45^. The ZIF-8 coating could effectively inhibit the recombination loss of the modified device. Later interfacial modification of the photoanode layer was demonstrated using the ZIF-8 layer to enhance both the short-circuit current and the open-circuit voltage^46^. Dou et al. presented a novel strategy for the application of MIL-125(Ti) for DSSCs. The hierarchically porous TiO_2_ nanocrystals prepared directly via the calcination of highly porous MIL-125(Ti) showed the high surface area and pore size with enhanced PCE owing to the superior dye adsorption, quick electron transport, and enhanced charge collection efficiency^47^.Hence to fabricate doped TiO_2_ nanostructures through MOF route is a highly promising strategy as these materials can avail the advantages of both doping and also the intrinsically unique structural properties inherited from the MOFs precursors^48,49^.

Here we report an efficient strategy to modify the photoanode of DSSCs using mesoporous cobalt doped TiO_2_ of unique morphologies derived from MIL-125(Ti) for the first time. The prepared doped TiO_2_ nanostructured TiO_2_ exhibited a PCE as high as 6.86% with a reduced bandgap. Extended visible light absorption was observed, which facilitated more absorbed photons as charge carriers with suppressed recombination rate. This simple method to modify the photoanode resulted in enhanced *J*_sc_ and PCE with reduced recombination loss, demonstrate the possibility of improving the next-generation DSSC photoanode to achieve promising device performance.

## Results and discussion

**Figure 1:**
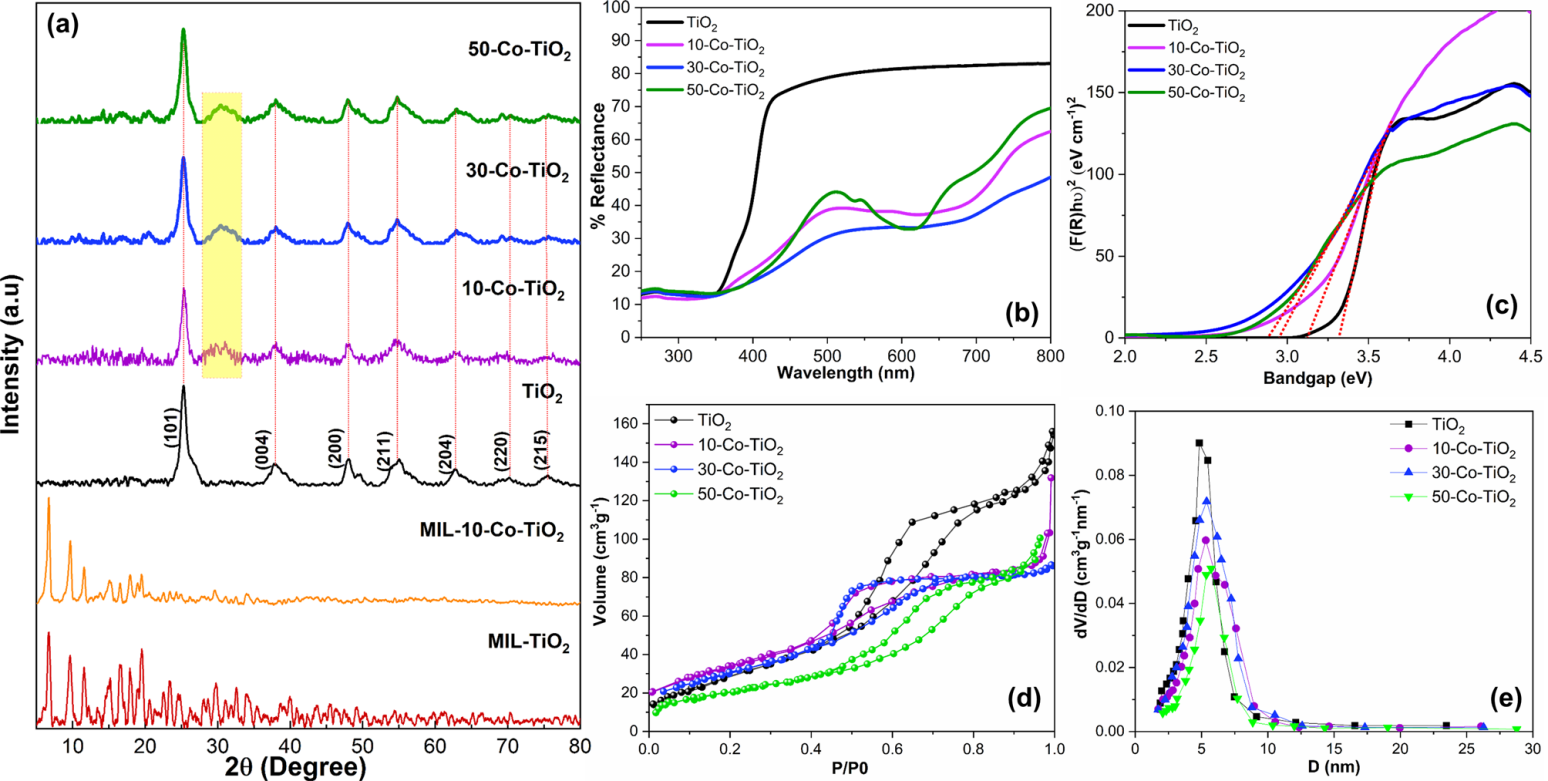
(**a**) PXRD patterns of pristine and doped MIL-125 (Ti) and the corresponding TiO_2_ semiconductor oxides after thermal annealing. (**b**) Diffuse reflectance spectra of MOF derived TiO_2_ samples and (**c**) its corresponding bandgap energy Kubelka–Munk plot (**d**) Nitrogen adsorption–desorption isotherms and (**e**) the corresponding BJH pore size distribution.

MIL-125(Ti) MOF was fabricated through a solvothermal process reported in previous literature^48^. Figure 1a shows the powder X-ray diffraction (PXRD) patterns of MIL-125(Ti), cobalt incorporated MIL-125(Ti), and the calcined MOF derived pristine and doped TiO_2_. Before the calcination process, the diffraction peaks obtained at 2θ = 6.7°, 9.5°, 9.7°, 11.5°, and 16.6° respectively; which are all in good agreement with those reported earlier for MIL-125 (Ti)^50,51^. After calcination, the samples showed crystalline peaks with the complete transformation of MOF precursor to pure metal oxide phase^52,53^. The observed diffraction peaks of calcined samples at 25.53°, 38.54°, 48.10°, 55.04°, 68.8° and 75.2° were indexed to (101), (004), (200), (105), (211), and (215) cubic crystal planes of anatase TiO_2_ according to the JCPDS card number 21-1272. The obtained XRD patterns of all cobalt doped samples almost coincide with that of pure anatase TiO_2_ without any peaks related to rutile or to metallic cobalt or cobalt oxide, endorsing that the anatase phase is not effected upon Co doping. A slight shift of the most intense (101) peak towards the lower 2θ values was identified for all doped samples, which is ascribed to be the expansion of the TiO_2_ unit cell due to the incorporation of Co^2+^. The ionic radius of Co^2+^ (Co^2+^  = 0.79 Å) is larger than that of Ti (Ti^4+^  = 0.745 Å) leading to an expansion TiO_2_ crystal with an increase of the lattice parameters. Also, a change in FWHM of (101) peak was observed after doping, which indicating the modification in the local structure around Ti^4+^ after Co doping. This confirms the successful incorporation of Co in TiO_2_ lattice. Apart from these, a small peak can be observed around 30.6° for the doped samples with the gradual increase in intensity (highlighted part in Fig. 1a). It is also inferred that the spectra obtained are devoid of any impurity during the solvothermal synthesis procedure. The obtained low-intensity peaks are attributed to the small size of the TiO_2_ nanoparticles formed.

The incorporation of cobalt into the TiO_2_ crystal lattice can influence the bandgap. This variation in bandgap after the cobalt incorporation can be clearly identified using diffuse reflectance spectroscopy, which is given in Fig. 1b. The full coverage of the UV–vis spectral range can be evident. In comparison to the pristine sample, Co^2+^ ion-doped TiO_2_ samples exhibited additional peaks in the visible region (450–800) in the sub-bandgap region. Doping has a direct influence on the optical properties of TiO_2_, which extended light absorption towards the visible region due to the formation of sub-bandgap states between the conduction and valence bands with oxygen vacancy sites^54^. The UV–vis diffuse reflection spectrum of pristine TiO_2_ is flat in the visible (400–800 nm) region. However, the reflection decreases drastically upon doping with cobalt^55^. The bandgap energy is determined from diffuse reflectance spectra following the theory of P. Kubelka and F. Munk presented in 1931 (Fig. 1c)^56^. Accordingly, a gradual decrease of the optical bandgap from ~ 3.3 to ~ 2.9 eV was obtained. The UV–Visible light response of the prepared samples was also studied by optical absorption spectroscopy of the samples in methanolic solution as in supplementary Figure S1. All of the doped samples showed much enhancement absorption compared to the pristine sample, which indicates the enhanced optical response of the samples after cobalt doping.

N2 adsorption–desorption isotherms were measured to investigate the surface area (Fig. 1d), and pore size distribution (Fig. 1e) of all samples. The samples showed type IV isotherm curves. The BET surface area and the pore volume of undoped TiO_2_ were obtained as 118 m^2^ g^−1^ and 0.32 cm^3^ g^−1^, respectively. A narrow pore size distribution around 5–6 nm was observed for TiO_2_ from the BJH analysis. All the doped samples showed slightly less surface area compared to undoped samples; 10-Co–TiO_2_ (92 m^2^ g^−1^and 0.30 cm^3^ g^−1^) 30-Co–TiO_2_ (90 m^2^ g^−1^and 0.33 cm^3^ g^−1^) and 50-Co–TiO_2_ (84 m^2^ g^−1^ and 0.29 cm^3^ g^−1^). Thus, the samples exhibit a relatively uniform mesoporous structure.

**Figure 2:**
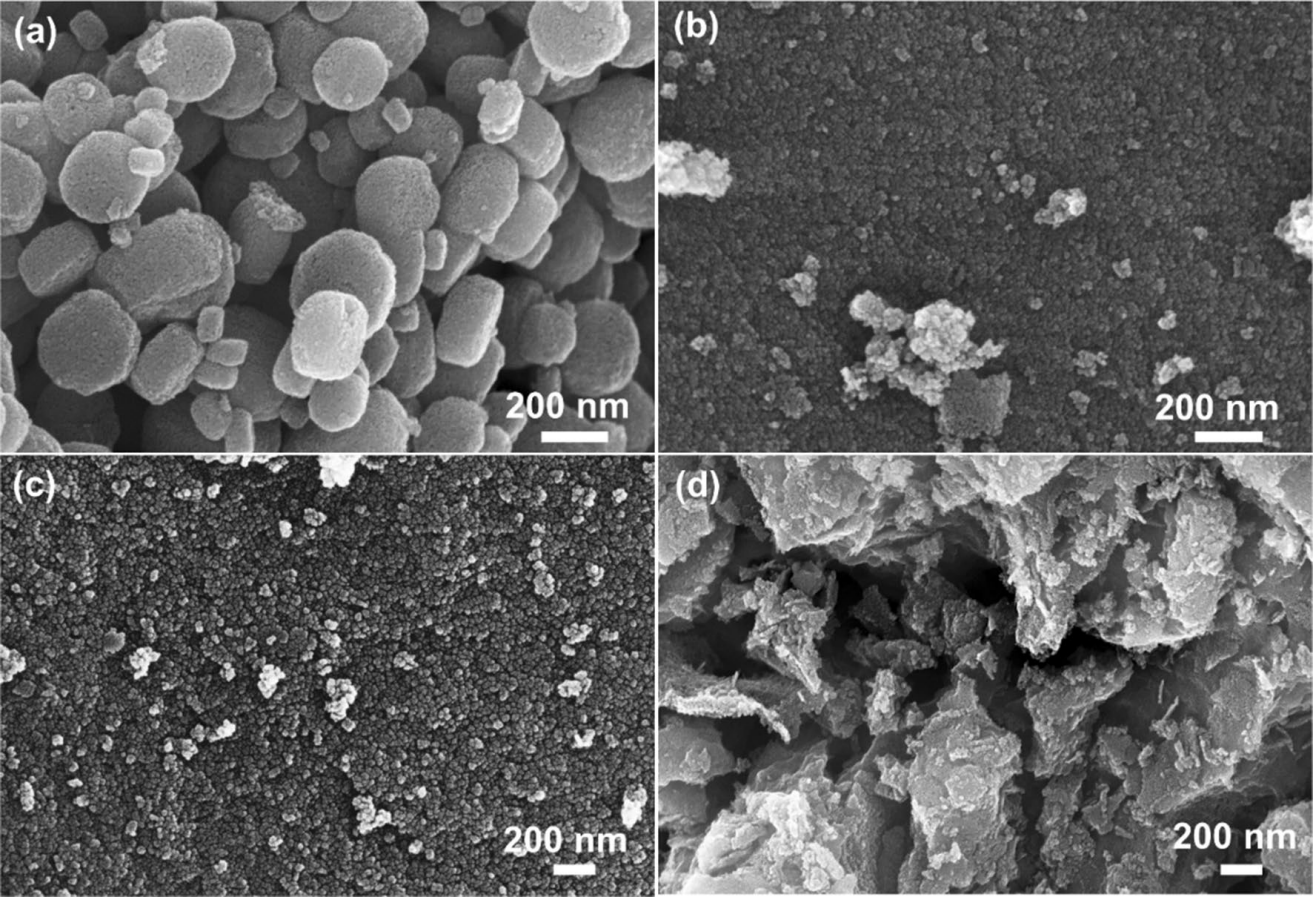
Morphological evolutions of MOF-derived TiO_2_ samples from nanodiscs to spherical nanoparticles on doping.

The morphological features of the sample before and after cobalt doping were characterized by SEM and TEM analysis. Figure 2a–d shows the SEM images of the doped and undoped samples. The undoped sample (Fig. 2a) depicted disc-like morphologies consisting of numerous nanoparticles with a diameter of 200–300 nm. Upon doping, the material showed drastic changes for both size and morphologies with very rough surfaces consisting of orderly arranged numerous nanoparticles of spherical shape with average sizes below 30 nm (Fig. 2b,c). At a higher doping concentration of cobalt, a more porous structured rough surface of the sample was perceived (Fig. 2d). The elemental identification of the sample was confirmed by EDS spectroscopy, and the spectra are given in supplementary Figure S2.

**Figure 3:**
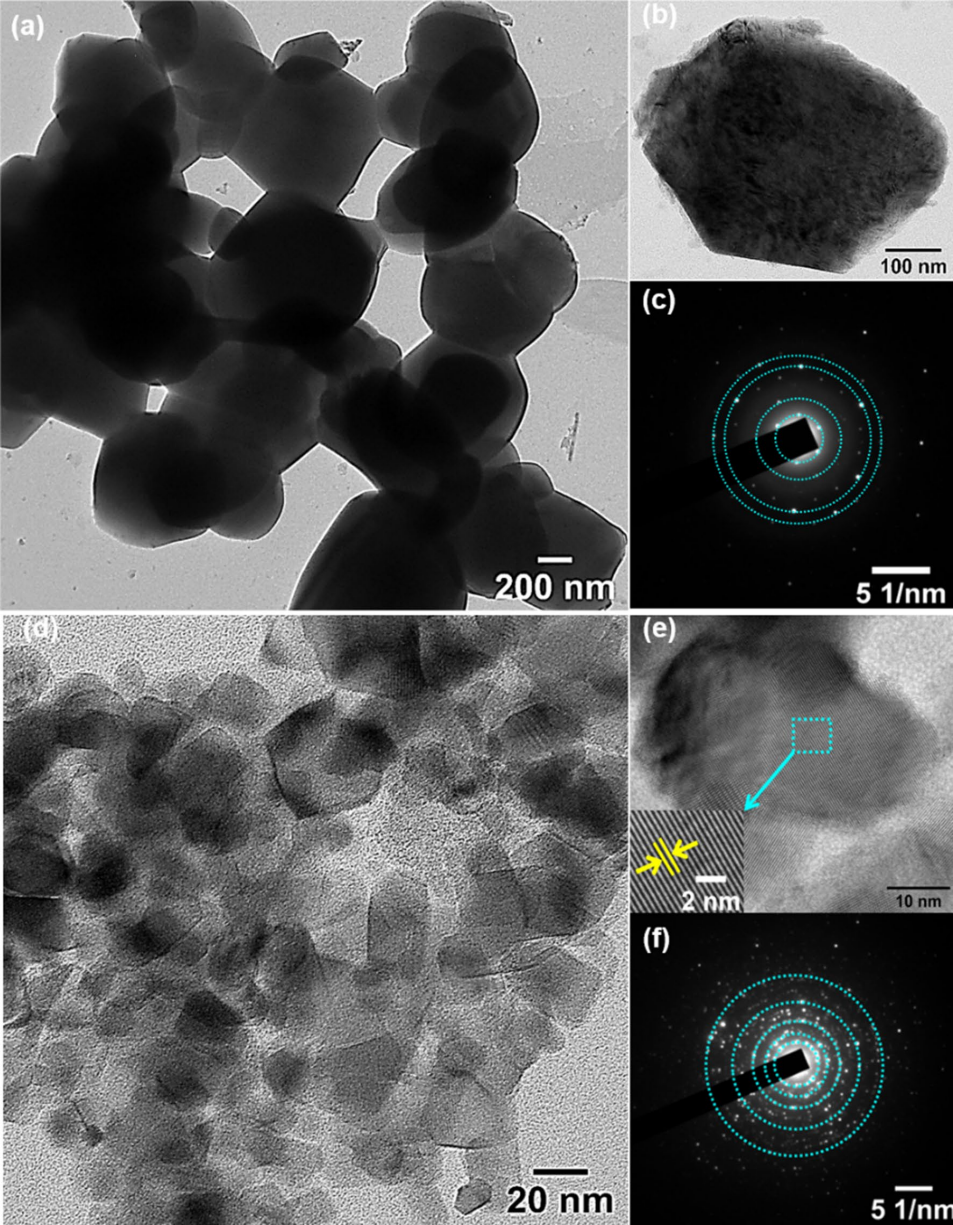
HR-TEM images of the MOF-derived pristine TiO_2_ and cobalt doped samples.

Further insights into the morphological and crystalline properties of samples were obtained from HR TEM analysis given in Fig. 3a–f. The pristine TiO_2_ particle was found to be of an average size of 0.5 µm (Fig. 3a,b). The SAED pattern (Fig. 3c) shows clear, distinct rings confirming the polycrystalline nature. The obtained rings in SAED pattern were indexed (101), (200), and (201) planes of tetragonal anatase TiO_2_ by calculating d-spacing. The result obtained is corroborated with the planes obtained in the XRD analysis of phase pure anatase TiO_2_. For undoped TiO_2_, the thickness of the particle observed was too high; as a result of this, the lattice fringes were not identified in HRTEM. However, the doped sample displayed (Fig. 3d) particle size of ~ 25 nm with clear lattice fringes as shown in Fig. 3e. HRTEM images displayed clear lattice fringes with d-spacing of 3.5 Å (inset figure of Fig. 3e) that matches (101) plane of tetragonal anatase phase of TiO_2_ ascertaining the preferable crystal growth plane is (101), that is corroborated with the maximum intense peak obtained in XRD^57^. Based on the FESEM and HR-TEM studies, the crystal growth mechanism can be explained. The formation of the precursor MOF (MIL125 (Ti)) occurred with eight TiO_6_ octahedra (oh) units attached by terephthalate linkers which results in the formation of a three-dimensional (3D) microporous network. When the solvothermal reaction proceeds, the solvent ions readily enter into the microporous channel and quickly react with MIL-125 (Ti) to form TiO_2_ nuclei and trigger the crystal growth process. During the crystal growth, the solvent ions prefer to adsorb on the (001) crystal faces of anatase TiO_2_. Since the [001] growth direction is inhibited, other growth directions such as [100] are enhanced, resulting in nano-disk morphologies of TiO_2_. A density difference occurs between MIL-125 (Ti) and TiO_2_ that can readily generate cracks in the MIL-125 (Ti) crystal. Thus the MOF precursor breaks into pieces. The presence of Co^2+^ ions affected the crystal growth pattern and resulted in a particle-like TiO_2_ NCs^58^. Upon increasing the concentration of dopants the agglomeration of NCs occurs which gives more open morphological structures made of small particle like NCs.

**Figure 4:**
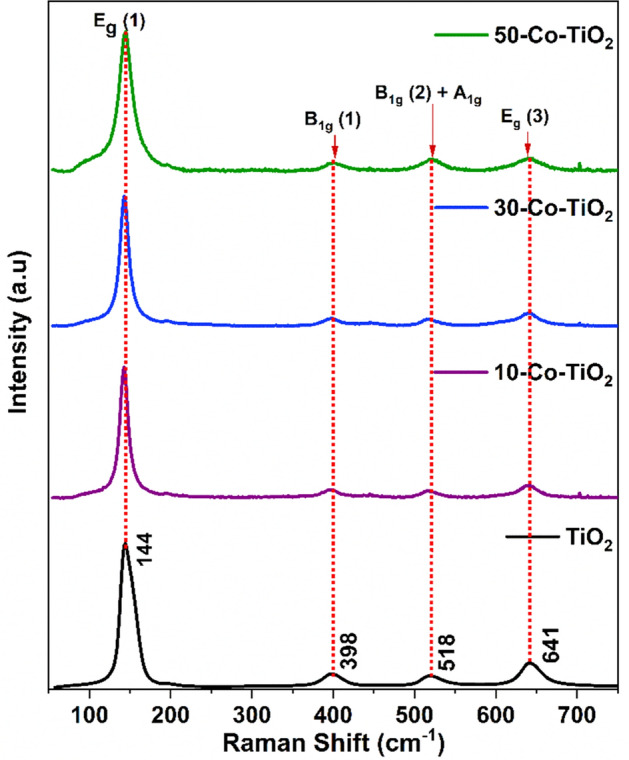
Room-temperature Raman spectra of pristine and doped TiO_2_.

Raman spectroscopic analysis was carried out to get more insight into the crystallinity, dopant-induced structural variations and also to understand the molecular vibration properties of all TiO_2_ samples, and the spectra are shown in Fig. 4. From group theory, anatase TiO_2_ has tetragonal structure and has six Raman active modes, A_1g_ + 2B_1g_ + 3E_g_, respectively^59^. E_g_ peak arises by O–Ti–O symmetric stretching vibration in TiO_2_, whereas B1g by O–Ti–O symmetric bending vibration and A_1g_ appears due to O–Ti–O anti-symmetric bending vibration. The peaks centered at 144, 398, 518, and 641 cm^−1^ are characteristic to E_g_(1), B_1g_, A_1g,_ and Eg modes, respectively. No Raman modes corresponding to the rutile phase were observed. The obtained spectra of the as-synthesized samples also showed the archetypal tetragonal form of phase pure anatase TiO_2_, which corroborates the obtained outcome from XRD. The intense E_g_(1) mode indicates the long-range order in the crystal. Upon doping with cobalt, the E_g_ (1) peaks of all doped samples show a blue shift and a gradual decrease in intensity. This peak broadening, blue shifting as well as weakening of peak intensity is owing to the non-stoichiometry created owing to oxygen vacancy and phonon confinement effect due to the nanoscale size effect of the crystallites. The breakdown of long-range translational crystal symmetry occurs by the incorporation of Co^2+^. As a result, oxygen vacancies were introduced, which results in the weakening of the Raman signals. Thus, the Raman results obtained were found to be dependable with the XRD data.

The thermal stability of the samples was studied using TGA analysis, and the resultant data are shown in supplementary Figure S3. Maximum weight loss was obtained for the MIL-TiO_2_ sample before calcination, which is possibly due to the existence of residual carbon present in the sample^60,61^. All the calcined samples (i.e., pristine and doped TiO_2_) showed significantly less weight loss as well as high thermal stability. Also, it is observed that there is almost no weight loss above 250 °C, signifying that all the organic moieties were removed after the annealing process. It is noteworthy to mention that a slight weight loss ~ 2 wt.% observed below 250 ◦C is due to water adsorption on the sample surface.

**Figure 5:**
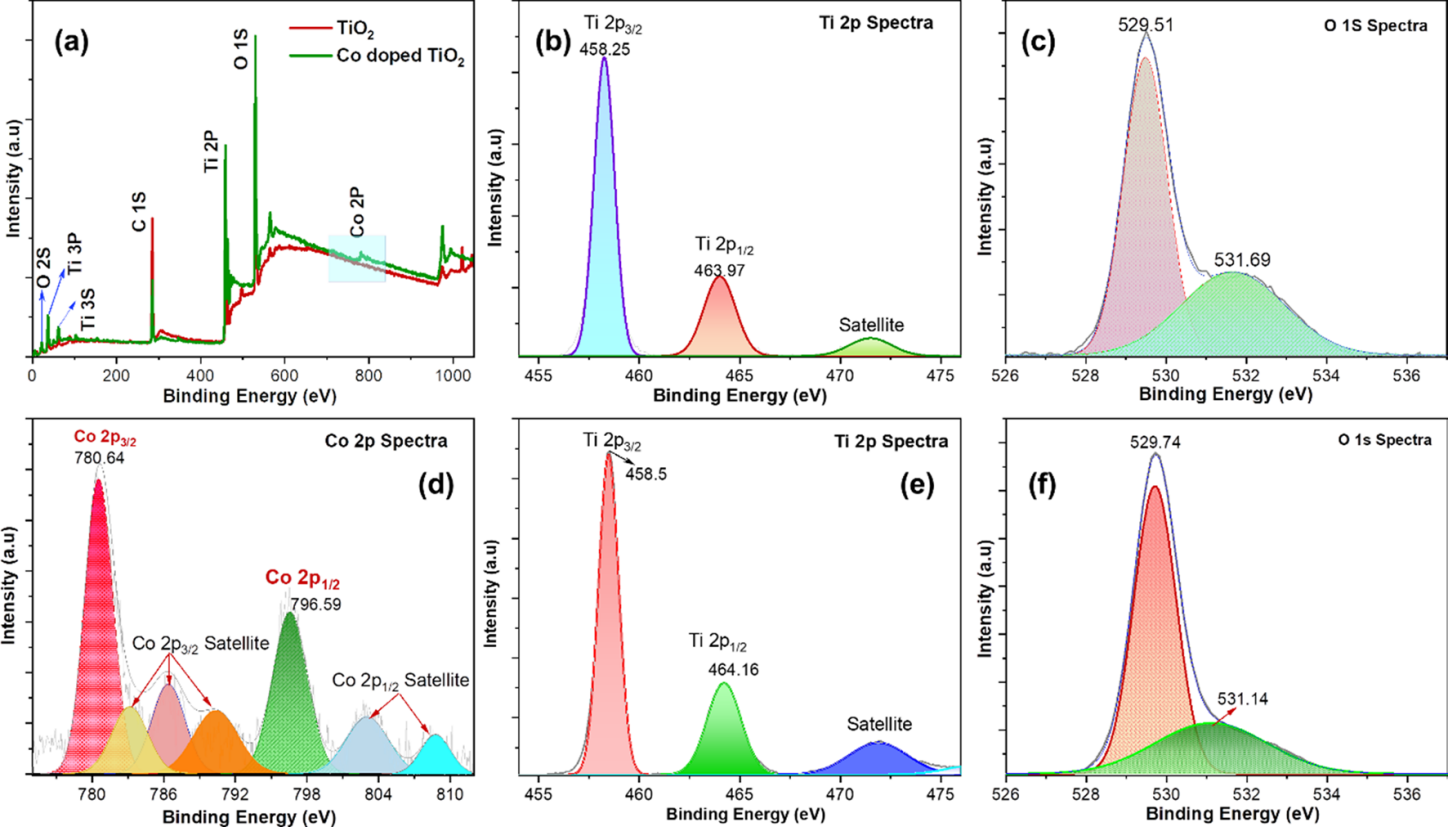
(**a**) A comparative XPS survey spectra of MOF derived TiO_2_ and cobalt doped TiO_2_ (**b**–**f**) Core spectra of Ti, O and Co.

The chemical composition, probability of secondary phases, oxygen vacancy, and oxidation state of surface elements were revealed by X-ray photoelectron spectroscopy (XPS) of the synthesized materials. Figure 5a–f shows the XPS spectrum of TiO_2_, and 30-Co–TiO_2_, respectively. Figure 5a is the survey spectrum of both the samples, which showed the characteristic peaks of Co, Ti, and O confirms the chemical composition in materials. The status of surface elements was further examined by the high-resolution XPS spectra; Fig. 5b presents the XPS spectrum of Ti 2p with Ti 2p_3/2_ and Ti 2p_1/2_ located at 458.25 and 463.97 eV, respectively, which matches the characteristic peaks of Ti^4+^–O bonds^62^.

From Fig. 5d of cobalt core-level spectra, peaks at binding energies of 780.64 eV of Co 2*p*_3/2_ are observed, which corresponds to the oxidation state of + 2 of cobalt in TiO_2_. Another peak observed around 796.59 eV corresponds to Co 2*p*_1/2_. The spin–orbit splitting value of 15.95 eV, as well as the peak broadening, clearly supports the successful inclusion of Co^2+^ over Ti atoms in the host lattice, that generates oxygen vacancy^63^. The presence of Ti^3+^ and Co^2+^ indicates that both samples have a definite amount of oxygen vacancies.

**Figure 6:**
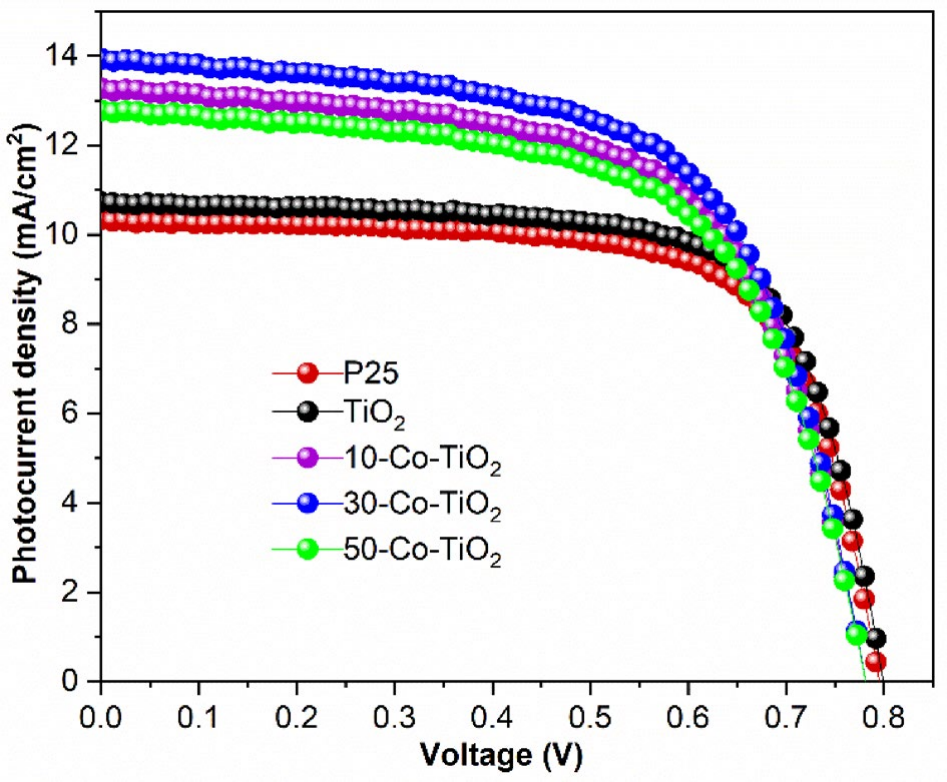
A comparative photocurrent density versus voltage curve (*J–V*) for MOF derived pristine TiO_2_ and Co^2+^ ion-doped TiO_2_ photoanode-based DSSCs.

DSSCs fabricated with undoped as well as Co^2+^ doped samples were analyzed under simulated sunlight AM 1.5 with power 100 mW cm^−2^. Figure 6 elucidates the *J–V* curves of the fabricated DSSCs. Table 1 summarizes the obtained parameters from the *J–V* curves, including short-circuit current density (*J*_*sc*_), open-circuit voltage (*V*_*oc*_), fill factor (FF), and power conversion efficiency (*PCE*). For the commercially available P25 titania sample, the obtained short circuit current and open-circuit voltage were *J*_*sc*_ = 10.35 mA/cm^2^, FF = 70.12% and*V*_*oc*_ = 0.79 V with a PCE of 5.76%.

**Table 1:** A comparative DSSC photovoltaic performance data determined by photocurrent density–voltage (*J–V*) and EIS characteristic analysis of different photoanodes.

Cell	*Jsc*	*Voc*	J_max_	V_max_	FF	PCE	R_S_	R_CT_ (Pt)	R_CT_ (TiO_2_)
	(mA/cm^2^)	(V)	(mA/cm^2^)	(V)	(%)	(%)	Ω/cm^2^	Ω/cm^2^	Ω/cm^2^
P25	10.35 ± 0.01	0.79 ± 0.01	8.91 ± 0.01	0.65 ± 0.01	70.13 ± 0.01	5.76 ± 0.01	20.36	0.86	14.72
TiO_2_	10.83 ± 0.02	0.80 ± 0.01	9.53 ± 0.04	0.64 ± 0.01	70.06 ± 0.01	6.06 ± 0.02	20.79	0.45	12.97
10-Co-TiO_2_	13.33 ± 0.01	0.79 ± 0.02	10.95 ± 0.01	0.59 ± 0.02	61.62 ± 0.02	6.46 ± 0.01	20.39	0.86	12.53
30-Co-TiO_2_	13.96 ± 0.01	0.78 ± 0.01	11.50 ± 0.02	0.60 ± 0.01	62.76 ± 0.01	6.86 ± 0.01	20.24	0.94	11.89
50-Co-TiO_2_	12.74 ± 0.03	0.78 ± 0.01	10.55 ± 0.01	0.59 ± 0.03	62.19 ± 0.01	6.19 ± 0.04	20.51	0.83	13.13

**Figure 7:**
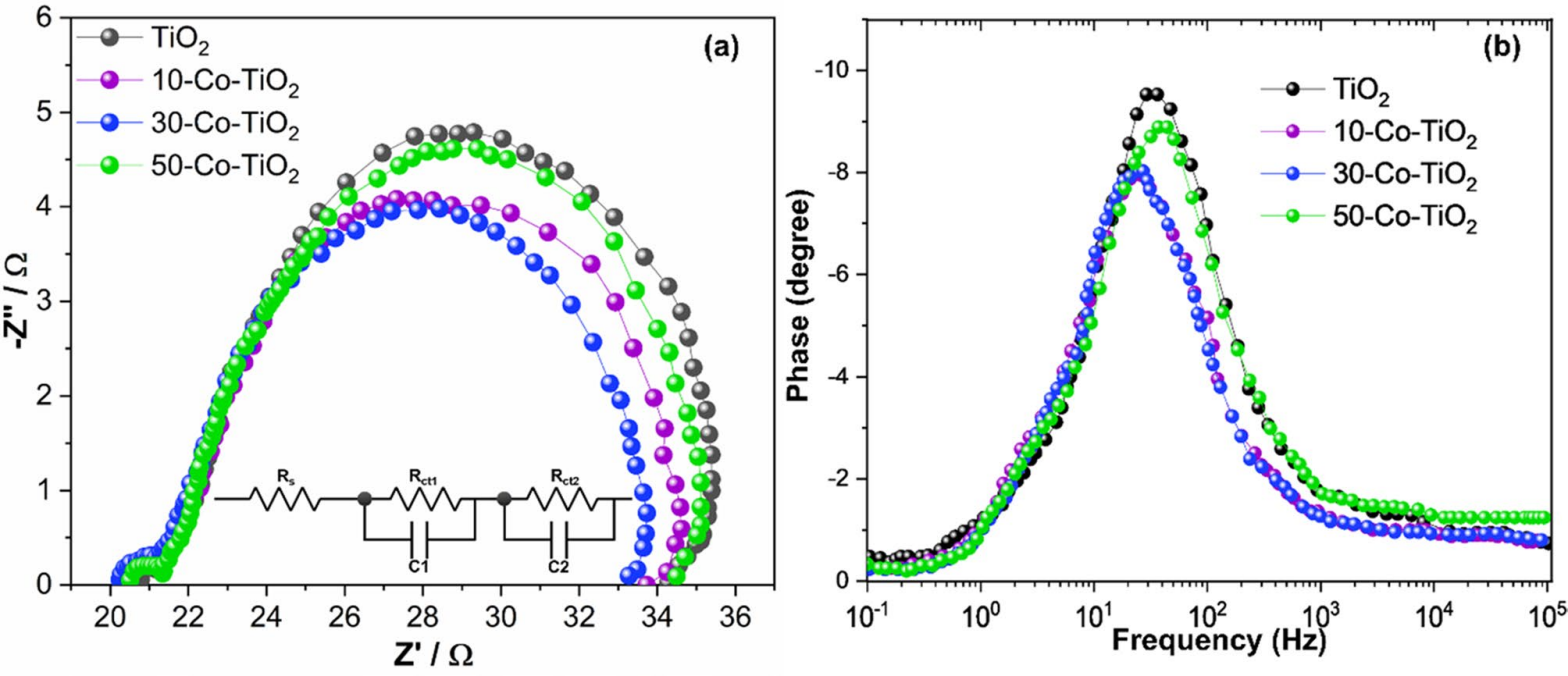
(**a**) Nyquist and (**b**) Bode impedance plot for TiO_2_ and different Co^2+^ ion doped TiO_2_ photoanode-based DSSCs. The equivalent circuit used to fit the impedance curve is inserted in (**a**).

**Figure 8:**
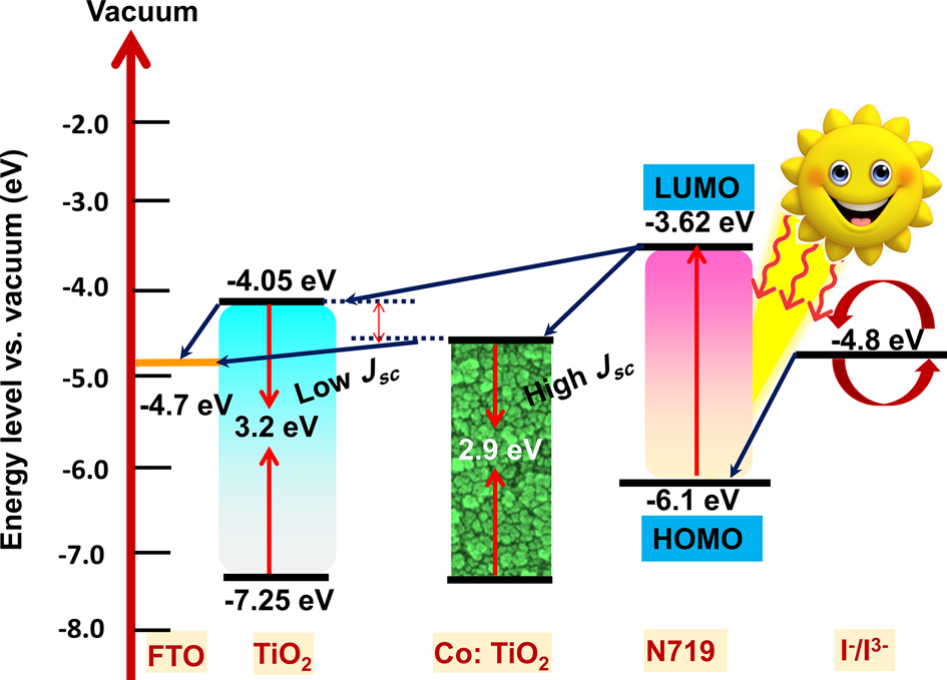
Schematics of possible electron transfer mechanism in MOF derived Co^2+^ ion doped TiO_2_photoanode based DSSCs.

MOF route-derived TiO_2_ showed enhanced PCE of 6.06% with improved *J*_*sc*_ and *V*_*oc*_ of 10.83 mA/cm^2^ and 0.799 V, respectively, however, the FF is reduced slightly to 70.05%Upon doping with 10 mg of cobalt, an increment in *J*_*sc*_ (of 13.33 mA/cm^2^) was observed while the *V*_*oc*_ (0.786) was decreased slightly. A considerable decrease in FF (61.62%) is observed. However, a net PCE of 6.46% was obtained, which showed a 12.3% improvement from the PCE data of fabricated DSSC with P25 as photoanode. When doping concentration was increased to 30 mg; PCE of 6.86% was achieved with increased *J*_*sc*_ = 13.96 mA/cm^2^, FF = 62.76% and slightly less *V*_*oc*_ = 0.78 V. Upon further increase of cobalt to 50 mg, *J*_*sc*_, FF and *V*_*oc*_ were decreased to 12.74 mA/cm^2^, 62.19% and 0.781 V with decreased PCE of 6.19%, but the PV performance was still greater in comparison to the pristine TiO_2_ sample. From the obtained results, it is understood that the FF of DSSCs fabricated with doped samples were decreased compared to the reference and pristine TiO_2_ based DSSCs. This can be explained as follows. The FF of a DSSC device mainly depends on the morphology and thickness of the photoanode film. Increased thickness of photoanode results in a high fill factor. However, the fill factor drops if the recombination of the charge carriers is more. Here, all the fabricated photoanodes in our study, including the reference photoanode, has the average thickness of 10–13 µm. Therefore, the difference in fill factor of DSSCs between the doped and reference photoanodes is due to the morphological difference, which can be clearly understood from the SEM and TEM characterization. Also, the BET studies showed the highly porous nature of the doped samples, which causes the excess of electrolyte diffusion into the photoanode film resulting in a high recombination rate of photogenerated electrons and subsequent reduction in FF^64, 65^. The fabricated DSSC cells were further analyzed to understand the electron lifetime and recombination characteristics with the help of the electrochemical impedance spectroscopic technique at a frequency range from 10^−1^ to 10^5^ Hz with an alternative current amplitude of 10 mV. The obtained Nyquist and Bode phase plots of pure and doped samples under AM 1.5 illumination were shown in Fig. 7a,b, respectively. In the Nyquist plot (Fig. 7a), two semicircles were obtained for all the samples. In which the small semicircle in the high-frequency region corresponds to the redox as well as electron transfer reaction at the Pt counter electrode–electrolyte interface^24^. The other large semicircle at the low-frequency region is related to the accumulation/transport of the photoinjected electrons within the photoanode film and the electron transfer across the TiO_2_/dye/electrolyte interface. This is considered as the characteristic peaks, which give a clear idea of the recombination behavior of electrons through the semiconductor oxide layer. The EIS curve is fitted using the equivalent circuit and the corresponding extracted parameters were given in Table 1. The sheet resistance (Rs) values obtained for all the devices were found to be similar and is ~ 20 Ω/cm^2^. The charge transfer resistance at the Pt CE interface obtained was below 1 Ω/cm^2^ for all the devices. The DSSCs fabricated using 30-Co–TiO_2_ based samples exhibited the lowest recombination resistance (11.89 Ω/cm^2^) compared to the reference (14.72 Ω/cm2) and undoped TiO_2_ (12.97 Ω/cm^2^) based DSSCs. The 10-Co–TiO_2_ and 50-Co–TiO_2_ based DSSC devices showed slightly higher resistance, 12.53 and 13.13 Ω/cm2, respectively. The result indicates the much-accelerated electron transfer in the device through reduction of charge transfer resistance resulting in an increment in *Jsc*. Figure 7b displays the Bode phase plots obtained from the impedance results. From Bode plots, the lifetime of the photo-injected electrons (τe) in semiconductor oxide layer can be calculated from the peak frequency (ƒmax) in the large semicircle region relate to the equation: τe = 1/2πƒmax; where ƒmax is the maximum characteristic peak frequency. The calculated τe values show an increased trend in a sequence of 30-Co–TiO_2_ (16.1 ms) > 10-Co–TiO_2_ (15.3 ms) > pure TiO_2_ (6.1 ms) > 50-Co–TiO_2_ (5.2 ms). In support of the Nyquist results, the 30-Co–TiO_2_ based cells demonstrated the lowest recombination resistance and the longest electron lifetime, which is accountable for the uninterrupted electron transport through the oxide layer to a longer distance, leading to the enhanced PCE^66,67^. However, at higher doping concentrations, the fabricated DSSCs revealed high recombination resistance at the semiconductor oxide/electrolyte interface with lesser τe (5.2 ms) which obstruct the cell performance by critically affecting the charge transport. On the other hand, at a lower doping concentration (10 mg of Co), the obtained τe was less (15.3 ms) as compared to 16.1 ms (for 10-Co–TiO_2_). Thus the impedance spectra are directly relating to the I–V results. Also, it is evident that doping can influence the morphology and also surface chemistry of the semiconductor film. In this work, maximum PCE was achieved for the doped sample with 30 mg Co^2+^, which was substantially ascribed to the improved surface roughness of the TiO_2_ after the successful integration of cobalt ions. Moreover, the additional cobalt ions in photoanode films can prevent effective photoinduced electron–hole pairs separation by acting as recombination centers, as evident from the EIS results. The particle-like morphologies induce better interconnection that bids appropriate deployment of incident light and fast electron transport. As the doping concentration increased to 50 mg, the resulted sample shows drastic changes in morphology with lots of voids and pores, resulting in the formation of secondary impurity phases as well the incorporation of Co ions at the interstitial position. Due to this, the device shows high recombination resistance with smaller τe. Based on the above observations, the deduced electron transport over the cobalt doped TiO_2_ photoanode is given in Fig. 8. Here the Co^2+^ doping in TiO_2_ significantly shifted the conduction band positively, which results in an enhanced driving force of electron injection and thus increases the electron injection efficiency from the LUMO of dye to the conduction band of TiO_2_. Thus, improved light absorption and repressed charge recombination is obtained, as obvious from the UV–vis and impedance spectroscopic measurements^68^. Thus, a significant improvement in *Jsc* was achieved. Doping could also protect the photoanode against photooxidation. Here the photon energy is effectively transferred to the Co^2+^ ions capable of quickly localizing the excitation and suppression of undesired reactions on the photoanode surface.

## Conclusion

In summary, we have successfully synthesized Co^2+^ ion-doped TiO_2_ nanocrystals via the MOF route. The structure and photovoltaic performance of the doped crystals were studied systematically using various characterization techniques. It was observed that the MOF-derived TiO_2_ samples have more surface roughness and more conductivity. The highest PCE of 6.86% in DSSC was achieved by 30 mg Co^2+^ ion-doped TiO_2_ in comparison to commercially available P25 Degussa (5.76%). The enhanced PCE was accredited to numerous factors, such as dopant-induced morphological and optical bandgap changes that resulted in improved light-harvesting efficiency with less recombination of photoinjected electrons. Better surface stability is attained after cobalt incorporation, which results in less dye aggregation on the surface with subsequent suppression in the recombination loss. The formation of several photoinduced charge carriers by charge transfer transition of Co^2+^ ions mainly contributed to the improved photocurrent, which paved the way for better PV performance. To further improve the PCE, modification of photoanode by the deposition of scattering layer and the study on its thickness is in progress.

## Materials and methods

N, N-dimethyl formamide (99.8%), DMF, cobalt acetate tetrahydrate (99.99%), terephthalic acid (98%), titanium (IV) butoxide (97%), and anhydrous ethanol (98%) were purchased from Sigma Aldrich. All the reagents were used intrinsically without additional purification, and deionized water was used for all the synthesis processes. All the solvothermal reaction was carried out in a 100mL autoclave provided by Amar Equipments Pvt. Ltd.

### Characterization

The synthesized catalyst samples were analyzed by powder X-ray diffraction (PXRD) technique using a Panalytical-Empyrean X-ray diffractometer, Netherlands with Cu-Kα radiation (λ = 1.54 Å) at 45 kV and 40 mA to scan the diffraction angles from 10° to 80°. The phase identification was carried out using standard patterns reported in the International Centre for Diffraction Data (ICDD) database. Thermogravimetric analysis (TGA) was carried out in a PerkinElmer, Simultaneous Thermal Analyzer (STA) from room temperature to 900 °C at 10 C/min under N_2_ atmosphere. FESEM and HR-TEM analysis were done using Tecnai G2 F20 instrument from FEI, Netherlands operating at 200KV. For TEM study, the samples were dispersed homogenously in isopropanol by ultrasonication and drop-casted onto a copper grid, and dried overnight before loading. Surface area and porosity measurements were analyzed using standard adsorption equipment AUTOSORB-1Q-MP-XR, Quantachrome Instruments, USA using N_2_ gas with 99.995% purity. The sample was activated under a vacuum at 150 °C for 3 h followed by nitrogen adsorption−desorption isotherm measurement at 77K. The surface composition and chemical states were measured with an Omicron Nanotechnology (Oxford Instruments) X-ray photoelectron spectroscope (XPS) equipped with monochromatic Al Kα radiation, and the obtained spectra were fitted by means of a Gaussian function. The peak correction was done with reference to the standard carbon 1s peak obtained at 285.07 eV. I–V analysis for the DSSC was recorded using the CHI660e from CH Instruments under one sun irradiation by PET Photo Emission Tech with SS50AAA solar simulator. Electrochemical Impedance spectroscopy (EIS) was measured by a Biologic potentiostat–galvanostat (SP-150) under an open circuit (*V*_*oc*_) under illumination. The frequency ranges from 0.1 Hz to 10^5^ Hz with 10 mV alternative (AC) signal.

### Synthesis:

#### MIL-125 synthesis

Ti-based MOFs (i.e., MIL-125) were synthesized by the solvothermal method. To a mixture solution of terephthalic acid (3 g), anhydrous methanol (6 mL), and anhydrous DMF (54 mL), butyl titanate (4.5 mM) was added slowly with continuous stirring. The whole mixture is loaded into a 100 mL teflon-lined stainless-steel autoclave and allowed to heat to 150°C for 24 hr. After, a white suspension of MIL-125 was obtained, separated by centrifugation, and washed with methanol (3 x 50 mL). The sample is subsequently dried in a vacuum oven at 80 °C for 12 h.

#### TiO_2_ and cobalt doped TiO_2_ synthesis

The as-synthesized MIL-125 sample was annealed at 400 °C at a rate of 2°C min^−1^ for 5 h to result in the pure titania powder stored in the sample vial for further uses. To prepare the doped TiO_2_ samples, CoCl_2_·6H_2_O (10, 30, and 50 mg) was added to the latter reaction mixture, and the same synthesis procedure was followed. After calcination, the doped samples were obtained with light to dark green depending on the amount of cobalt precursor.

#### Photoanode preparation

To Prepare the DSSC photoanode, the synthesized samples (0.5g) were dispersed into 4mL ethanol by ultrasonication for 15 minutes. Then add 1.5g terpineol and 0.10g ethyl cellulose and sonicate the solution to form a homogenous viscous paste. The prepared paste was deposited on cleaned fluorine-doped tin oxide (FTO) glass substrate (transparent electrode with a typical square sheet resistance of 10 Ω/sq and overall transmittance about 85% in the visible range) by doctor blade method to get a thickness of ~13–15 μm using 3M scotch tape into an area of 0.25 cm^2^ (with proper masking). After drying in the air, the coated substrates were heated in a furnace (450°C for 2h). After reaching room temperature, the substrates were immersed in a 0.5 mM solution of N719 dye (Sigma-Aldrich) for 30 minutes.

#### Counter electrode preparation

To prepare the Pt-counter electrodes, one or two drops of 5 mM H_2_PtCl_6_ solution in isopropanol were drop cast onto the FTO and subsequently sintered at 400°C for 20 min. The transparent electrode obtained was used as such as the counter electrode without further heat treatment or modification.

#### Electrolyte preparation and assembling of DSSC

The photoanode and counter electrode were sandwiched together with surlyn film of 30 μm thickness as a spacer. Further, one or two drops of redox couple electrolyte consisting of 0.06 M 1-butyl-3-methyl imidazolium iodide, 0.03 M of iodine solution (I_2_), 0.10 M guanidinium thiocyanate, 0.5 M 4-tert-butylpyridine dissolved in a mixture of acetonitrile and valeronitrile (volume ratio, 85:15), were added and introduced into the DSSC with a syringe.

## Supplementary Information

Supplementary Information.
