# Supplementary material for: MOF-derived Co2+-doped TiO2 nanoparticles as photoanodes for dye-sensitized solar cells
Source: Sci Rep. 2021 Aug 11;11:16265. doi: 10.1038/s41598-021-95844-4 (PMC8358052; doi:10.1038/s41598-021-95844-4)
Supplement: Supplementary file 1 — Supplementary Information. [file 41598_2021_95844_MOESM1_ESM.docx]

**Electronic Supplementary Information**

**MOF-Derived Co^2+^-Doped TiO_2_ Nanoparticles as Photoanodes for Dye-Sensitized Solar Cells**

R. Krishnapriya ^1,2^, C. Nizamudeen^1^, B. Saini^2^, A.S.M. Mozumder^3^, Rakesh K. Sharma^2^, and A-H. I. Mourad ^1,4,5*^

**Note: This supplementary information contains supplementary Figures S1-S3 and Table S1**

**Figure S1.** UV-Visible light response of the synthesized samples in methanol solution.


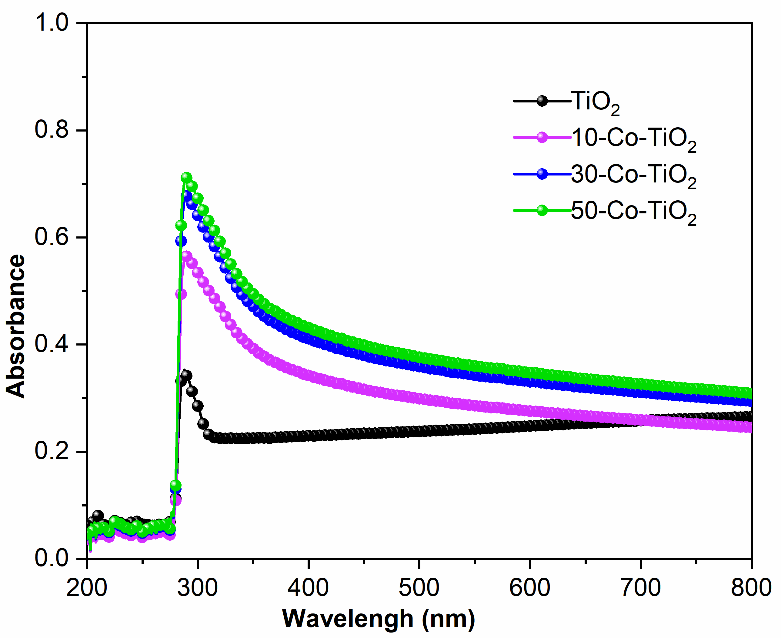


**Figure S2.** EDS spectra of MOF derived (a) TiO_2_ (b) 10-Co- TiO_2_ (c) 30-Co- TiO_2_ (d) 50-Co-TiO_2_

**
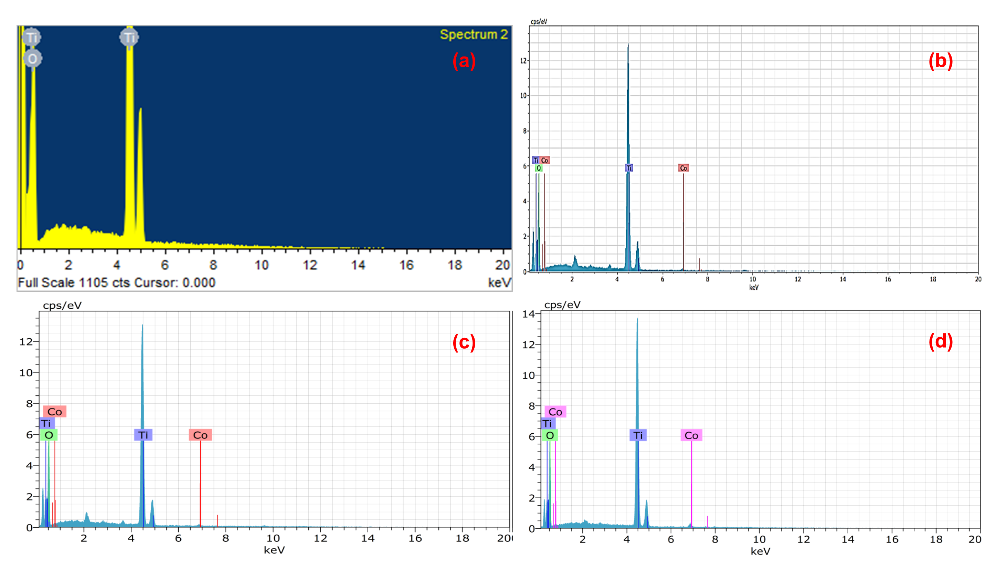
**

**
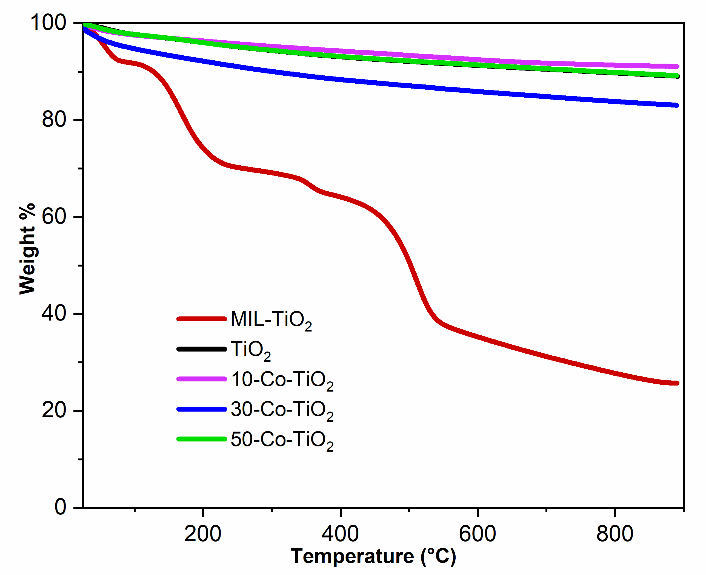
**

**Figure S3.** TGA curves of prepared samples.

**Table S1.** A comparative DSSC photovoltaic performance data determined by photocurrent density-voltage (*J–V)* Characteristics of five fabricated devices each for various photoanode materials.

| **Cell** | ***Jsc*** | ***Voc*** | **J_max_** | **V_max_** | **FF** | **PCE** |
| --- | --- | --- | --- | --- | --- | --- |
|  | ***(mA/cm^2^)*** | ***(V)*** | ***(mA/cm^2^)*** | ***(V)*** | **(%)** | **(%)** |
| **P25-1** | 10.358 | 0.8042 | 8.9176 | 0.657 | 70.1359 | 5.7732 |
| **P25-2** | 10.343 | 0.7892 | 8.9026 | 0.642 | 70.1209 | 5.7582 |
| **P25-3** | 10.338 | 0.7842 | 8.8976 | 0.637 | 70.1159 | 5.7532 |
| **P25-4** | 10.348 | 0.7942 | 8.9076 | 0.647 | 70.1259 | 5.7632 |
| **P25-5** | 10.353 | 0.7992 | 8.9126 | 0.652 | 70.1309 | 5.7682 |
| **TiO_2_-1** | 10.8472 | 0.809 | 9.5695 | 0.646 | 70.0691 | 6.0808 |
| **TiO_2_-2** | 10.8172 | 0.794 | 9.5095 | 0.631 | 70.0541 | 6.0508 |
| **TiO_2_-3** | 10.8072 | 0.789 | 9.4895 | 0.626 | 70.0491 | 6.0408 |
| **TiO_2_-4** | 10.8272 | 0.799 | 9.5295 | 0.636 | 70.0591 | 6.0608 |
| **TiO_2_-5** | 10.8372 | 0.804 | 9.5495 | 0.641 | 70.0641 | 6.0708 |
| **10-Co-TiO_2_-1** | 13.3397 | 0.806 | 10.962 | 0.6095 | 61.6418 | 6.4662 |
| **10-Co-TiO_2_-2** | 13.3247 | 0.776 | 10.947 | 0.5795 | 61.6118 | 6.4512 |
| **10-Co-TiO_2_-3** | 13.3197 | 0.766 | 10.942 | 0.5695 | 61.6018 | 6.4462 |
| **10-Co-TiO_2_-4** | 13.3297 | 0.786 | 10.952 | 0.5895 | 61.6218 | 6.4562 |
| **10-Co-TiO_2_-5** | 13.3347 | 0.796 | 10.957 | 0.5995 | 61.6318 | 6.4612 |
| **30-Co-TiO_2_-1** | 13.9747 | 0.793 | 11.52 | 0.6067 | 62.7668 | 6.8721 |
| **30-Co-TiO_2_-2** | 13.9597 | 0.778 | 11.49 | 0.5917 | 62.7518 | 6.8571 |
| **30-Co-TiO_2_-3** | 13.9547 | 0.773 | 11.48 | 0.5867 | 62.7468 | 6.8521 |
| **30-Co-TiO_2_-4** | 13.9647 | 0.783 | 11.5 | 0.5967 | 62.7568 | 6.8621 |
| **30-Co-TiO_2_-5** | 13.9697 | 0.788 | 11.51 | 0.6017 | 62.7618 | 6.8671 |
| **50-Co-TiO_2_-1** | 12.7508 | 0.791 | 10.56 | 0.6164 | 62.2021 | 6.2265 |
| **50-Co-TiO_2_-2** | 12.7298 | 0.776 | 10.545 | 0.5714 | 62.1871 | 6.1665 |
| **50-Co-TiO_2_-3** | 12.7228 | 0.771 | 10.54 | 0.5564 | 62.1821 | 6.1465 |
| **50-Co-TiO_2_-4** | 12.7368 | 0.781 | 10.55 | 0.5864 | 62.1921 | 6.1865 |
| **50-Co-TiO_2_-5** | 12.7438 | 0.786 | 10.555 | 0.6014 | 62.1971 | 6.2065 |

---------------------------------
